# Characteristics of gastric fluid microbiota in patients with refractory *Helicobacter pylori* infection

**DOI:** 10.3389/fmicb.2025.1618803

**Published:** 2025-07-01

**Authors:** Huiting Zhu, Shijie Sun, Yujing Wang, Wensi Zhang, Shiyu Du, Yanli Zhang

**Affiliations:** ^1^Department of Gastroenterology, China-Japan Friendship Hospital, Beijing, China; ^2^Clinical Research Institute of China-Japan Friendship Hospital, Beijing, China; ^3^State Key Laboratory of Biochemical Engineering, Institute of Process Engineering, Chinese Academy of Sciences, Beijing, China; ^4^Key Laboratory of Biopharmaceutical Preparation and Delivery, Chinese Academy of Sciences, Beijing, China

**Keywords:** refractory *helicobacter pylori* infection, gastric fluid microbiota, 16S rRNA gene sequencing, microbial diversity, microbial interactions

## Abstract

**Introduction:**

Refractory *Helicobacter pylori* infection (RHPI) poses a clinical challenge due to its treatment resistance. The gastric microbiota characteristics in patients with RHPI remain unclear. This study analyzed gastric fluid samples to explore the structural and functional differences in the gastric microbiota of patients with RHPI and their association with clinicopathological features, providing a theoretical basis for precision treatment of RHPI.

**Methods:**

Eighty-four patients who underwent gastroscopy were prospectively and consecutively divided into the Nhp (*H. pylori*-negative, *n* = 32), Php (*H. pylori*-positive at first diagnosis without treatment, *n* = 32), and Rhp (RHPI, *n* = 20) groups. Gastric fluid and mucosal biopsy samples were collected for 16S rRNA gene sequencing, pathological evaluation, and bioinformatic analysis. Differences in gastric microbiota and clinical data were compared among the three groups.

**Results:**

The Rhp group exhibited more gastric mucosal atrophy, inflammation, and inflammatory activity than Nhp and Php groups. Rhp also showed lower microbial richness and diversity. *β*-diversity analysis revealed distinct microbial communities between the Nhp and Php/Rhp groups. Rhp was enriched with *H. pylori* PZ5004 and *Pseudoalteromonas* sp. C_8, among others, while Php was enriched with *Lactobacillus* sp. CY1 and other species and Nhp with *Prevotella melaninogenica* and other species. The Rhp group also had higher *H. pylori* PZ5004/P79 abundance, more complex microbial interactions, and enriched sulfur relay pathways than the other groups.

**Conclusion:**

*H. pylori* infection disrupts the diversity, structure, and function of gastric microbiota. The close interaction between characteristic microbiota and *H. pylori* subspecies may be a key factor contributing to the difficulty in treating RHPI.

## 1 Introduction

*Helicobacter pylori* (*H. pylori*) infection is a major cause of gastritis, peptic ulcers, gastric MALT lymphoma, and gastric cancer (Malfertheiner et al., 2023). Eradicating *H. pylori* is the primary preventive strategy against these diseases. Eradication therapy has successfully reduced the global *H. pylori* infection rate from 58.2 to 43.1% (Chen et al., 2024). However, the prevalence of refractory *H. pylori* infection (RHPI) is increasing annually, presenting a major challenge owing to antibiotic resistance, poor medication adherence, and insufficient acid suppression. RHPI is defined as the persistence of *H. pylori* infection after two or more standardized eradication treatments (Shah et al., 2021).

Recent studies have highlighted the presence of complex microbial communities in the stomach, which are implicated in disease onset and progression. In 2006, Bik et al. (2006) utilized 16S rDNA sequencing to demonstrate the diversity of the gastric mucosal microbiota. Research using INS-GAS mice has further shown that co-existing bacteria can enhance *H. pylori*-induced gastric cancer (Lofgren et al., 2011; Lertpiriyapong et al., 2014; He et al., 2022). For example, Fu et al. (2024) confirmed that *Streptococcus anginosus* can promote gastric cancer development.

Several studies have described the characteristics of the gastric mucosal microbiota at various pathological stages, revealing distinct microbiota compositions associated with different stages of disease progression (Vasapolli et al., 2019; Li et al., 2021; Nikitina et al., 2023; Jin et al., 2024). Patients with RHPI may harbor a unique gastric microbiota composition, which could play a critical role in the persistence of *H. pylori* infection. However, the specific structure and function of this microbiota remain poorly understood, as few studies have investigated its characteristics.

The gastric mucosa is commonly used for gastric microbiota analysis in research, but it has some limitations. Collecting mucosal samples is an invasive procedure, and the limited sample size may not allow to fully capture the characteristics of the entire gastric microbiota. In contrast, the gastric fluid, which is in prolonged contact with the mucosa, can provide a more comprehensive and accurate representation of the gastric microbiota. Gastric fluid collection for microbiota detection and analysis is a relatively simple, safe, and non-invasive method. Several studies have demonstrated that analyzing the gastric fluid in different disease states can reveal variations in the structural composition of microbial environments (Li et al., 2022; You et al., 2024).

This study used 16S rRNA gene sequencing to compare the gastric fluid microbiota among *H. pylori*-negative individuals, *H. pylori*-positive patients at first diagnosis without treatment, and patients with RHPI to understand the microbiota characteristics in RHPI and their association with clinicopathological features. The goal was to provide a new theoretical basis and approaches for improving RHPI treatment.

## 2 Materials and methods

### 2.1 Study population

Patients who underwent gastroscopy at the Endoscopy Center of the China-Japan Friendship Hospital between February 2023 and October 2023 were included in this study. This study was approved by the Clinical Research Ethics Committee of the China-Japan Friendship Hospital (Ethics Review Number: 2023-KY-116), and all participants provided signed informed consent.

Inclusion criteria: (1) adults aged 18–75 years, regardless of sex, who voluntarily participated and cooperated in the study; (2) diagnosis of *H. pylori* infection based on the results of the ^13^C urea breath test, gastric mucosal histological staining, and rapid urease test; (3) the interval since the last *H. pylori* eradication therapy was more than 6 months.

Exclusion criteria: (1) use of antibiotics, probiotics, proton pump inhibitors, H_2_ receptor antagonists, potassium-competitive acid blockers, bismuth compounds, hormones, immunosuppressants, or other relevant medications within 4 weeks prior to sample collection; (2) presence of serious underlying diseases; (3) diagnosis of malignant tumors in the digestive tract; (4) a history of gastric or esophageal surgery.

Grouping criteria: patients were grouped based on the results of three diagnostic tests (^13^C urea breath test, gastric mucosal histological staining, and rapid urease test). (1) Nhp group: patients were assigned to the non-*H. pylori* (Nhp) group if two of the three diagnostic tests were negative, and there was no history of *H. pylori* eradication treatment; (2) Php group: patients were assigned to the positive *H. pylori* (Php) group if two of the three diagnostic tests were positive, and there was no history of *H. pylori* eradication treatment; (3) Rhp group: patients were assigned to the refractory *H. pylori* (Rhp) group if two of the three diagnostic tests were positive, and they had failed two or more standardized *H. pylori* eradication treatments.

### 2.2 Sample collection and 16S rRNA gene sequencing

All participants underwent gastroduodenal endoscopy after a 12-h fasting period, performed by a specialized endoscopist. The gastric mucosal condition was recorded according to the Kimura–Takemoto Classification. During the procedure, 10–15 mL of clear gastric fluid was aspirated and collected into a sterile container; then, it was rapidly transported on ice for storage at −80°C for long-term preservation. Routine mucosal biopsy samples were obtained from the lesser curvature of the antrum for pathological examination. The gastric mucosal samples were stained with hematoxylin and eosin, and pathological diagnoses were made by two professional pathologists according to the updated Sydney system.

The Kimura–Takemoto classification criteria are as follows: C-type (closed type): C1, characterized by atrophy limited to the antrum; C2, atrophy affects the gastric angle or lower body; C3, atrophy is confined to the upper area of the lesser curvature; O-type (open type): O1, characterized by the atrophic border lying between the lesser curvature and the anterior wall; O2, defined by atrophy affecting the anterior wall of the gastric body; O3, marked by atrophy spanning the entire stomach, resulting in the absence of folds along the greater curvature. Based on endoscopic observation, the degree of atrophy is categorized as mild (C1, C2), moderate (C3, O1), or severe (O2, O3) (Tziatzios et al., 2024).

The updated Sydney system is used to perform pathological evaluation based on five dimensions: inflammation grade, inflammation activity, glandular atrophy, intestinal metaplasia, and dysplasia.

For the gastric fluid microbiota analysis, 16S rRNA gene sequencing was performed. Total genomic DNA was extracted from gastric fluid samples using the CTAB/SDS combined lysis method. For the V3–V4 hypervariable region of the 16S rDNA gene, specific primers were used for polymerase chain reaction (PCR) amplification. The forward primer was 515F (5′-GTGCCAGCMGCCGCGGTAA-3′), and the reverse primer was 806R (5′-GGACTACHVGGGTWTCTAAT-3′). PCR products were electrophoresed and then purified. Subsequently, the TruSeq® DNA PCR-Free Kit (Illumina, USA) was used for library construction, and high-throughput sequencing was conducted on the Illumina NovaSeq 6,000 platform (PE150 mode). Raw data were converted into the FASTQ format using bcl2fastq v2.20 software, followed by quality control with Trimmomatic (v0.39) to remove low-quality sequences and adapter contamination. High-quality sequence data were assembled using USEARCH (v11.2.64) to obtain high-quality tagged data.

### 2.3 Bioinformatics and statistical analyses

The tagged sequences from all samples were clustered using the UPARSE-OTU algorithm (97% similarity threshold), generating operational taxonomic units (OTUs). Species annotation was performed using the Ribosomal Database Project (RDP v16_2021) database, and the SINTAX algorithm (confidence threshold of 0.8) was applied for classification to identify microbial species within the samples. The Chao1 and Shannon diversity indices were used to assess microbial richness and evenness, reflecting the *α*-diversity of the samples. Principal coordinate analysis was performed based on a Bray–Curtis distance matrix and weighted UniFrac distances to describe the differences in microbial communities among different sample groups and for visualization.

Linear discriminant analysis effect size (LEfSe) was used to identify microbial species differences between groups, highlighting taxonomic units significantly associated with each group. Taxa with linear discriminant analysis >2 and false discovery rate-adjusted *p* ≤ 0.05 were considered to have differing abundance features. Functional prediction of the gut microbiota was performed using the Tax4Fun (v0.3.1) tool, based on the SILVA_138_SSURef_Nr99 database, to predict and analyze the KEGG metabolic functions of the microbiota.

Statistical analysis was performed using SPSS v26.0. Continuous variables are expressed as mean ± SD, while categorical variables are presented as frequencies and percentages. Differences between groups for continuous variables were analyzed using a one-way analysis of variance or the Kruskal–Wallis test. Categorical variables were compared using the chi-squared test or Fisher’s exact test. All statistical tests were two-sided; a *p*-value <0.05 was considered statistically significant.

## 3 Results

### 3.1 Patient clinical characteristics

A total of 84 participants were included in the study, all of whom completed the collection of gastric fluid and mucosal samples. There were 32, 32, and 20 patients in the Nhp, Php, and Rhp groups, respectively. There were no statistically significant differences in age or sex among the three groups. The general clinical data of the patients were analyzed; a comprehensive evaluation of these findings is presented in Table 1.

**Table 1:** Clinical baseline data and pathological characteristics of gastric mucosa among the three groups.

Evaluated Parameters	Nhp (*n* = 32)	Php (*n* = 32)	Rhp (*n* = 20)	*p*-value
Mean age ± SD, years	43.81 ± 2.21	40.69 ± 1.08	44.00 ± 2.63	0.46
Sex: Male [*n* (%)]	12 (37.50)	18 (56.25)	6 (30.00)	0.13
Kimura–Takemoto classification system				<0.01
CSG	32	23	8	Nhp-Php < 0.01
CAG (Mild)	0	6	9	Nhp-Rhp < 0.01
CAG (Moderate)	0	3	2	Php-Rhp = 0.03
CAG (Severe)	0	0	1	
Updated Sydney system				
Inflammation grade				<0.01
Mild	29	5	1	
Moderate	3	22	15	Rhp-Php = 0.34
Severe	0	5	4	
Inflammatory activity				<0.01
Absent	30	2	0	
Mild	2	11	1	
Moderate	0	19	16	Rhp-Php<0.01
Severe	0	0	3	
Atrophy				0.11
Absent	29	30	15	
Present	3	2	5	Rhp-Php = 0.06
Intestinal metaplasia				0.14
Absent	27	24	12	
Present	5	8	8	Rhp-Nhp = 0.21
Dysplasia				0.26
Absent	31	29	20	
Present	1	3	0	Rhp-Nhp = 0.16

Nhp, *Helicobacter pylori*-negative group; Php, *H. pylori*-positive at first diagnosis without treatment group; Rhp, refractory *H. pylori* infection group; SD, standard deviation; CAG, chronic atrophic gastritis; CSG, closed-type superficial gastritis.

The Kimura–Takemoto classification system was used to assess the range and degree of gastric mucosal atrophy. Under the system, the Rhp group exhibited a greater extent of gastric mucosal atrophy than did the Nhp and Php groups, indicating more severe atrophy with statistically significant differences (*p* < 0.01).

The updated Sydney system was used to evaluate the severity of gastritis, including parameters such as mucosal inflammation, atrophy, intestinal metaplasia, and dysplasia. Under the updated Sydney system for the antral mucosa, both the Rhp and Php groups showed more severe mucosal inflammation than the Nhp group (*p* < 0.01); however, no significant difference in mucosal inflammation grade was observed between the Rhp and Php groups. Statistically significant differences in inflammation activity were observed among the three groups (*p* < 0.01). No significant differences were observed in the incidences of glandular atrophy, intestinal metaplasia, and dysplasia across the groups.

### 3.2 Gastric fluid microbiota analysis

#### 3.2.1 Rarefaction curves

As the sequencing depth increased, the rarefaction curves for all samples gradually reached a plateau, indicating that the sequencing depth was sufficient to cover most microbial species present and met the analytical requirements (Supplementary Figure 1).

#### 3.2.2 Gastric fluid microbiota structure

The compositions of the three groups at the phylum and genus levels are shown in Supplementary Figures 2A,B.

#### 3.2.3 Microbial diversity analysis

**Figure 1:**
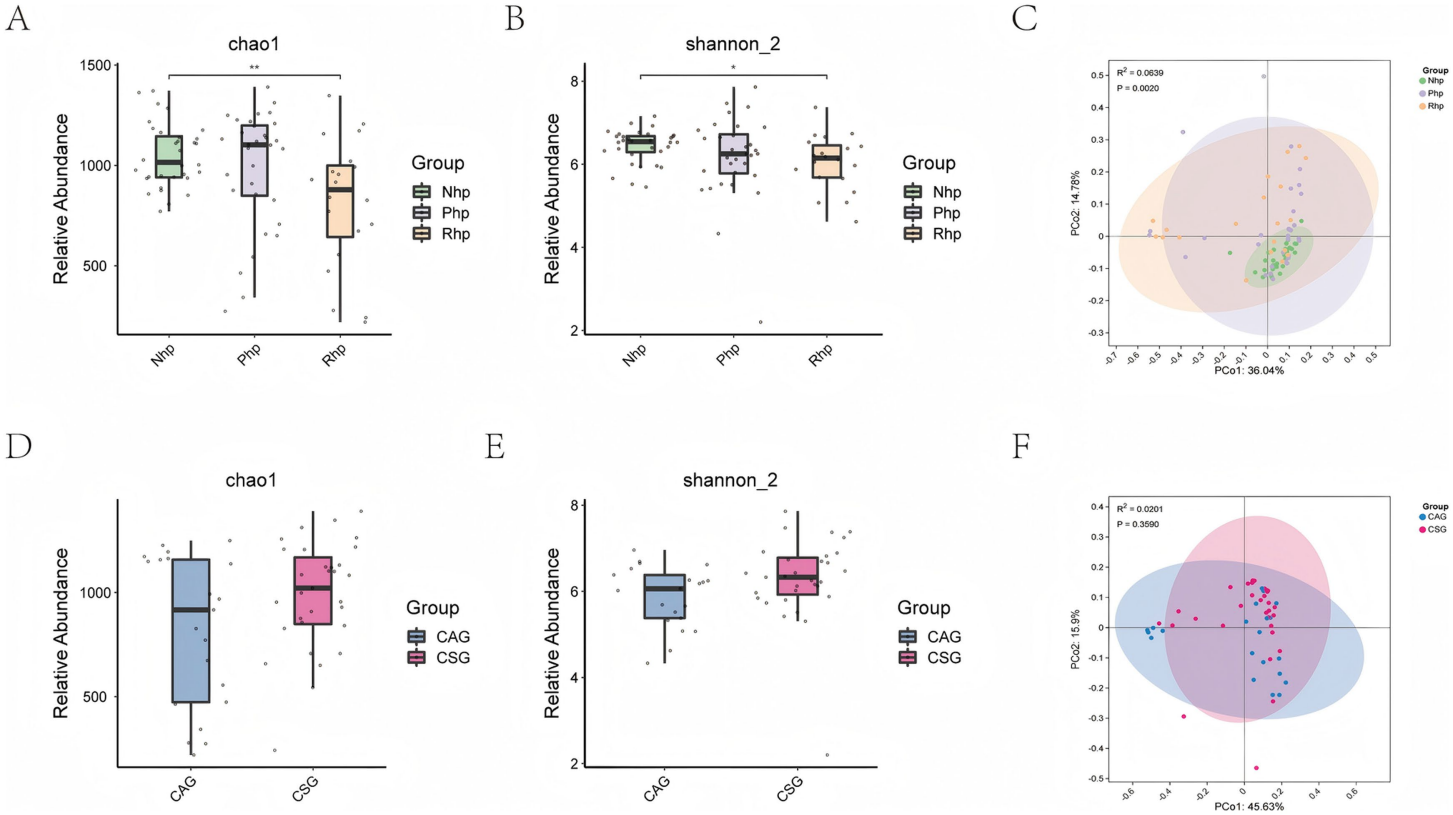
Comparison of *α*-diversity and *β*-diversity between groups. **(A)** Chao1 index comparison between the three groups; **(B)** Shannon index comparison between the three groups; **(C)** PCoA plot of microbial community structure among the three groups; **(D)** Chao1 index comparison between *Helicobacter pylori*-infected patients with and without mucosal atrophy; **(E)** Shannon index comparison between *H. pylori*-infected patients with and without mucosal atrophy; **(F)** PCoA plot of microbial community structure in *H. pylori*-infected patients with and without mucosal atrophy. PCoA, Principal Coordinate Analysis plot; Nhp, *H. pylori*-negative group; Php, *H. pylori*-positive at first diagnosis without treatment group; Rhp, refractory *H. pylori* infection group; CAG, chronic atrophic gastritis; CSG, closed-type superficial gastritis.

Microbial diversity, which reflects species richness and diversity, was assessed using the Chao1 (1048.35 ± 156.64 vs. 986.87 ± 298.32 vs. 812.78 ± 317.32) and Shannon_2 (6.43 ± 0.41 vs. 6.16 ± 1.01 vs. 6.05 ± 0.65) indices (Figures 1A,B). The *α*-diversity indices of the Rhp group were significantly lower than those of the Nhp group (Chao1 index *p <* 0.01; Shannon_2 index *p* = 0.02). However, there were no statistically significant differences in the *α*-diversity indices between the Php group and Nhp or Rhp groups despite the Php group being infected with *H. pylori*.

Principal component analysis was used to describe differences in the microbial community structure between the three groups (Figure 1C). The Nhp group exhibited a distinct microbial community structure compared to both the Php and Rhp groups. Samples within the Nhp group showed high similarity in community structure. The Php and Rhp groups shared similar microbial community structures, with no statistically significant differences between them.

Further exploration of the gastric fluid microecological structure in patients with and without mucosal atrophy under endoscopy in the context of *H. pylori* infection revealed no significant differences in microbial richness, diversity, or community composition between the two groups (Figures 1D–F).

#### 3.2.4 LEfSe analysis

**Figure 2:**
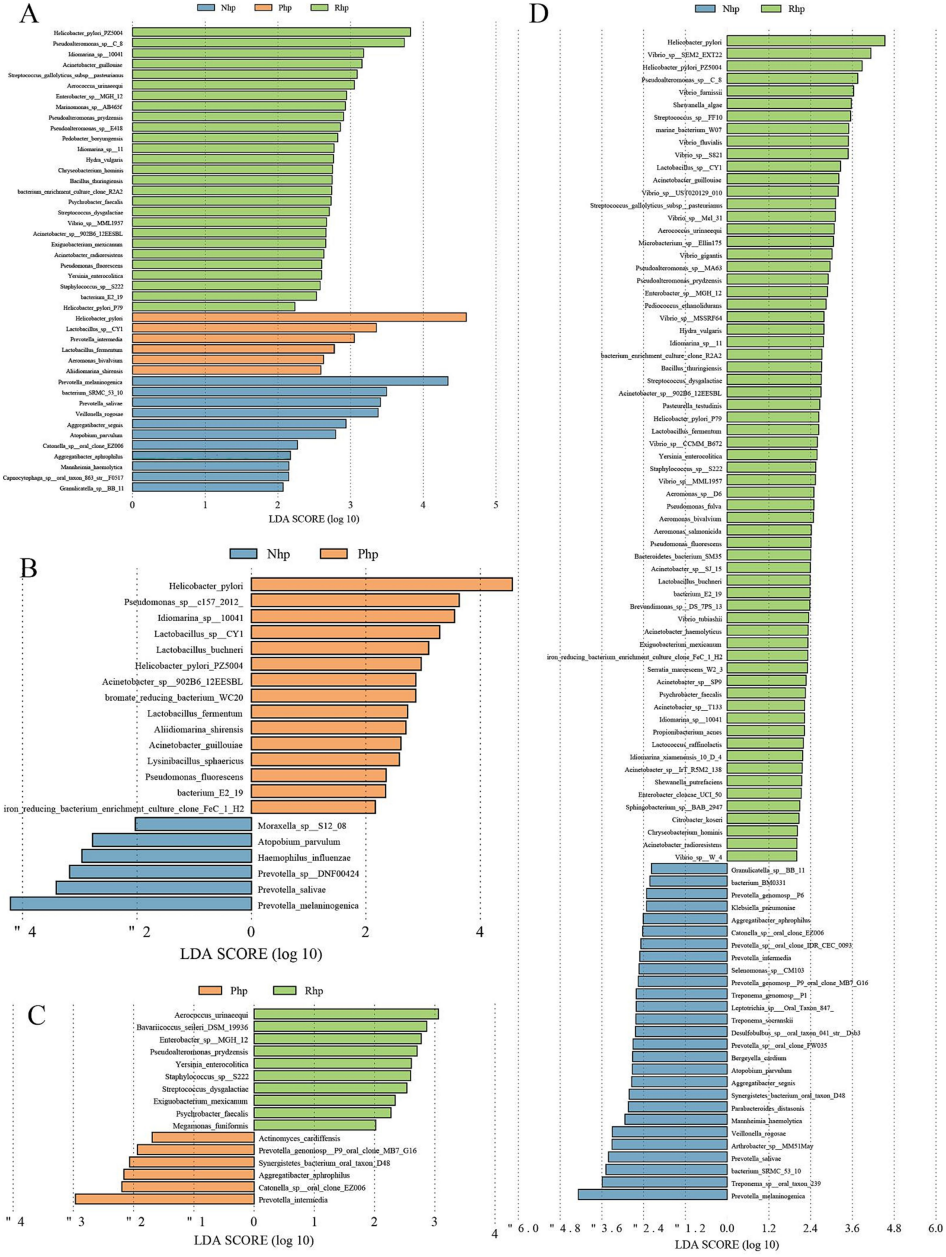
Linear discriminant analysis plots using linear discriminant analysis effect size analysis. **(A)** Comparison among the three groups; **(B)** Comparison between the Nhp and Php groups; **(C)** Comparison between the Php and Rhp groups; **(D)** Comparison between the Nhp and Rhp groups. Nhp, *Helicobacter pylori*-negative group; Php, *H. pylori*-positive at first diagnosis without treatment group; Rhp, refractory *H. pylori* infection group.

LEfSe analysis was performed to identify differentially abundant taxa among the three groups (Figure 2). A total of 44 differential microorganisms were identified across the groups. The linear discriminant analysis revealed that *H. pylori* PZ5004, *Pseudoalteromonas* sp. C_8, *Idiomarina* sp. 10,041, *Acinetobacter guillouiae*, *Streptococcus gallolyticus sub*sp. *pasteurianus*, *Aerococcus urinaeequi*, and 21 other microorganisms were enriched in the Rhp group. *H. pylori*, *Lactobacillus sp.* CY1, *Prevotella intermedia*, *Lactobacillus fermentum*, *Aeromonas bivalvium*, and *Aliidiomarina shirensis* were enriched in the Php group. *Prevotella melaninogenica*, bacterium SRMC_53_10, *Prevotella salivae*, *Veillonella rogosae*, and eight other microorganisms were enriched in the Nhp group.

Further analysis of *H. pylori* in the Rhp and Php groups revealed that *H. pylori* PZ5004 and *H. pylori* P79 were significantly more abundant in the Rhp group than in the Php group.

#### 3.2.5 Microbial interaction network

**Figure 3:**
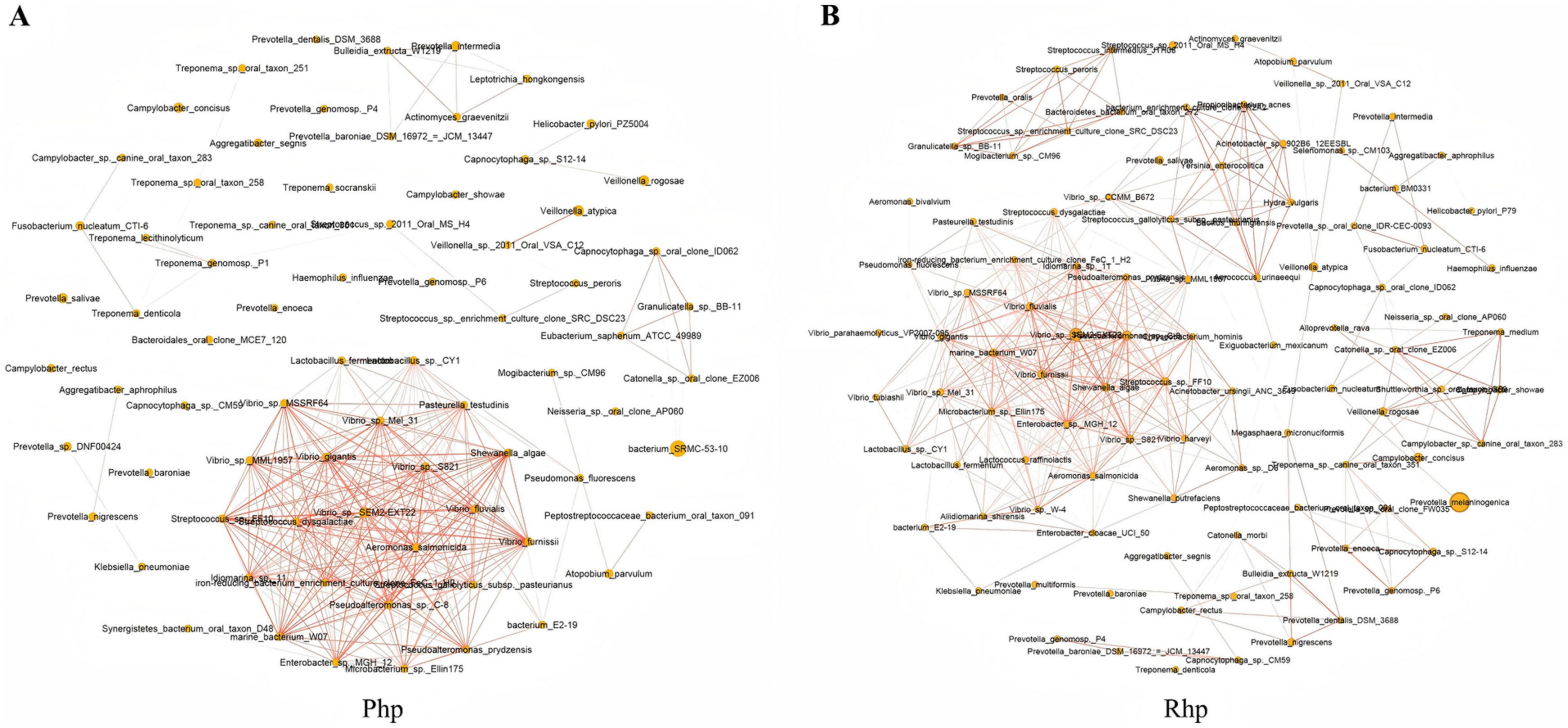
Bacterial interaction network diagrams for the Php group **(A)** and the Rhp group **(B)**. Php, *Helicobacter pylori*-positive at first diagnosis without treatment group; Rhp, refractory *H. pylori* infection group.

The microbiome interaction networks for the Php and Rhp groups are shown in Figures 3A,B. Differences in core density, key species roles, and co-occurrence patterns of marginal microorganisms were observed between the groups.

In the Php group, the core area of the network was dense, with numerous co-occurrence relationships among microorganisms, including *Vibrio*, *Pseudoalteromonas*, *Lactobacillus*, and *Streptococcus*, which exhibited relatively dense connections. Similarly, the core area of the Rhp group was also dense. However, in the Rhp group, additional connections were formed among many marginal species. The overall network in the Rhp group demonstrated more complex lines, and the distribution of connections across some nodes was more uniform than that in Php group.

#### 3.2.6 16S rRNA gene functional metabolic prediction

**Figure 4:**
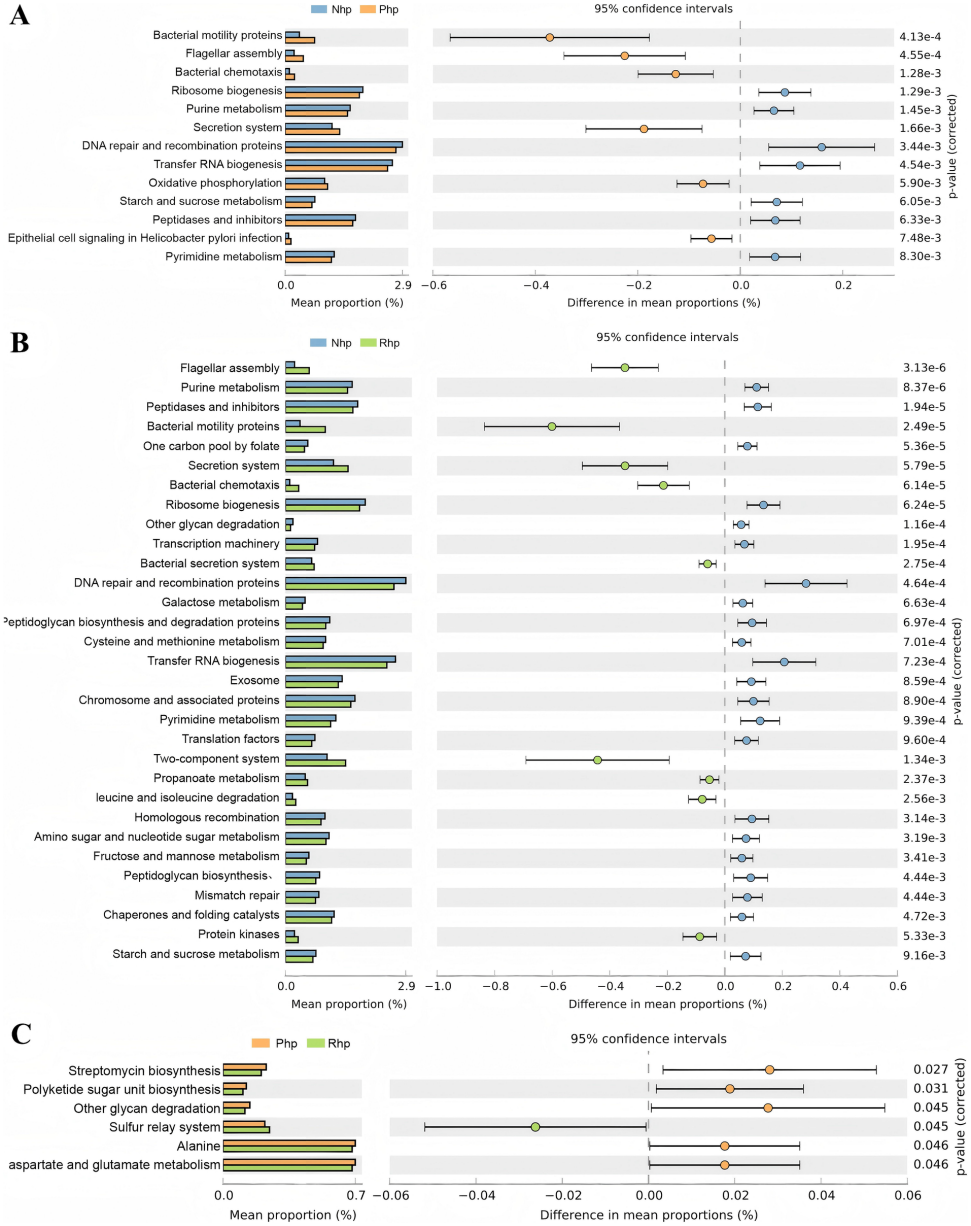
Differential functional prediction analysis of gastric fluid microbiota among the three groups. **(A)** Comparison between the Nhp and Php groups; **(B)** Comparison between the Nhp and Rhp groups; **(C)** Comparison between the Php and Rhp groups. Nhp, *Helicobacter pylori*-negative group; Php, *H. pylori*-positive at first diagnosis without treatment group; Rhp, refractory *H. pylori* infection group.

Functional metabolic predictions for the 16S rRNA genes in the three groups were performed using the Phylogenetic Investigation of Communities by Reconstruction of Unobserved States (PICRUSt2) tool, with annotations and comparisons based on the KEGG database. The results showed that six metabolic pathways were enriched in the Php group, including bacterial motility proteins, flagellar assembly, bacterial chemotaxis, secretion systems, oxidative phosphorylation, and epithelial cell signaling in *H. pylori* infection (Figure 4A).

In the Rhp group, nine metabolic pathways, including those related to flagellar assembly, bacterial motility proteins, secretion systems, bacterial chemotaxis, bacterial secretion systems, two-component systems, propanoate metabolism, leucine and isoleucine degradation, and protein kinases, were enriched (Figure 4B). When comparing the Rhp and Php groups, only one metabolic pathway—the sulfur relay system—was enriched (Figure 4C).

## 4 Discussion

Research indicates that 1–3% of individuals infected with *H. pylori* eventually develop gastric cancer, and approximately 89% of gastric cancer cases are attributable to *H. pylori* infection (Malfertheiner et al., 2023). *H. pylori* has been classified as a class I carcinogen by the World Health Organization, highlighting the clinical significance of its eradication therapy. With the annual increase in cases of RHPI, its treatment has emerged as an important clinical challenge (Shah et al., 2021).

The gastrointestinal microbiota plays a critical role in human health, and alterations in microbial composition may contribute to the development of various diseases. The gastric microbiota environment is particularly complex, and it remains uncertain whether structural changes in the microbiota influence the success of *H. pylori* eradication. In this study, we analyzed the gastric fluid microbiota as well as the endoscopic and pathological features of the gastric mucosa in patients categorized as *H. pylori*-negative, primary *H. pylori*-positive, and RHPI. Our findings demonstrated that *H. pylori* infection disrupted the normal microbial diversity, structural composition, and gastric function, with RHPI being associated with more pronounced alterations.

Using the Kimura–Takemoto classification and the updated Sydney system, we analyzed the extent of gastric mucosal atrophy and pathological findings. Although both the Rhp and Php groups had *H. pylori* infection, the extent of gastric mucosal atrophy in the Rhp group was significantly greater than that in the primary Php group (*p* < 0.05). Furthermore, the level of mucosal inflammation was more severe in the Rhp group (*p* < 0.05). We hypothesized that this may be related to potential genetic mutations in *H. pylori* strains in patients with RHPI, as these strains may carry more or stronger virulence factors that damage the structure and function of gastric mucosal cells and enhance the inflammatory response (Ansari and Yamaoka, 2019). Conversely, repeated use of antibiotics for eradication therapy may disrupt the gastric microbiota, thereby compromising the host’s mucosal barrier and making the gastric mucosa more susceptible to damage (Huang et al., 2022).

Previous studies have reported no significant difference in the *α*-diversity between *H. pylori*-negative individuals and those infected with *H. pylori* (Chen et al., 2023; Huang et al., 2024). Our findings aligned with these observations, showing that the richness and diversity of the gastric microbiota in patients positive for *H. pylori* infection at initial diagnosis were lower than those in *H. pylori*-negative individuals, although the difference was not statistically significant. However, we found that the α-diversity of the gastric microbiota in patients with RHPI was significantly lower than that in both *H. pylori*-negative and *H. pylori*-positive patients at initial diagnosis.

Interestingly, regarding the *β*-diversity, *H. pylori*-negative patients exhibited a more stable structure than both *H. pylori*-positive patients and patients with RHPI, with significant statistical differences. These differences may be attributed to the previous use of antibiotics during eradication therapy, which likely reduced the richness and diversity of the gastric microbiota. Furthermore, the prolonged colonization of *H. pylori* may have disrupted the gastric microenvironment, impairing the growth and colonization of normal microbiota.

Previous studies have suggested that as the pathological stage progresses, the characteristics of the mucosal microbiota undergo gradual changes, leading to a distinct microbiota structure (Ferreira et al., 2018; Sung et al., 2020). In RHPI, changes in microbial diversity may result from *H. pylori* infection itself or the prolonged duration of infection, which progressively alters the pathological and physiological structure of the mucosa. However, the evolution of secondary microflora remains unclear. To investigate this, we examined the gastric microbiota in patients with and without mucosal atrophy during *H. pylori* infection. Although our findings indicated that patients with mucosal atrophy had slightly lower *α*-diversity, the difference was not statistically significant. Therefore, we hypothesize that *H. pylori* infection may exert a greater influence on the gastric microbiota structure than on the pathological state itself.

LEfSe analysis identified 44 differential microbial taxa across the three groups, suggesting that, beyond antibiotic resistance, the presence of specific bacterial species may contribute to the difficulty in eradicating *H. pylori* infection. Compared to *H. pylori*-positive patients at initial diagnosis, patients with RHPI showed enrichment of two *H. pylori* subspecies, PZ5004 and P79, in the stomach. The genotypes of these subspecies may harbor unique virulence factors, potentially contributing to antibiotic resistance and more severe mucosal damage, warranting further investigation. These findings support the notion that, in addition to antibiotic resistance, multistrain infections may complicate *H. pylori* eradication—a hypothesis corroborated by previous studies (Huang et al., 2024).

Interestingly, *Lactobacillus* was not detected in the gastric fluid of patients with RHPI. Research suggests that *H. pylori* infection leads to an increase in *Lactobacillus* abundance (Iino et al., 2018). We speculate that during initial *H. pylori* infection, *Lactobacillus* may proliferate as an adaptive response, playing a protective anti-inflammatory role by altering the microbiota structure. However, in the Rhp group, repeated rounds of eradication therapy with antibiotics likely eliminated *Lactobacillus*. This aligns with the findings of Liu et al. (2022), who observed *Lactobacillus* enrichment in patients with an initial *H. pylori* infection but its absence in patients with RHPI. Similarly, studies have shown that probiotics such as *Bifidobacterium* and *Lactobacillus* can serve as effective adjunctive treatments for *H. pylori* infection (Efrati et al., 2012; Poonyam et al., 2019; Dash et al., 2024). Based on this evidence, we hypothesize that incorporating *Lactobacillus* into RHPI eradication therapy could enhance eradication rates by optimizing gastric anti-inflammatory capacity.

Additionally, we observed enrichment of *Serratia*, *Acinetobacter*, and *Streptococcus* in the Rhp group. *Streptococcus* includes several subspecies that produce various toxins and are involved in suppurative inflammation, hypersensitivity, and immune responses. Studies have confirmed the gastric carcinogenicity of *Streptococcus* spp. in pharyngitis (Fu et al., 2024). Therefore, the higher level of mucosal inflammation observed in patients with RHPI may be linked to the accumulation of *Streptococcus*, although the specific mechanism warrants further investigation.

The analysis of the bacterial interaction network revealed that certain microorganisms associated with inflammation or intestinal diseases, such as *Vibrio*, exhibited a higher frequency of coexistence and stronger ecological influence or functional association in *H. pylori*-positive environments. In contrast, the gastric microecological interaction in RHPI was significantly altered, resulting in a more complex ecological interaction network. This observation aligns with the LEfSe analysis, which showed that *Streptococcus* was enriched in patients with RHPI. We hypothesize that the complexity of microbial interactions could be a contributing factor to the difficulty in treating RHPI. A tightly connected microbiome and a more intricate network of interactions may contribute to the stability of the microbiome structure but hinder the eradication of *H. pylori*. However, our findings differ from those of previous studies. Chen et al. (2023) found that microbial interactions in the mucosal microbiota of patients were reduced. This discrepancy may be attributed to differences in biological sample sources, as their study used gastric mucosal samples, while ours used gastric fluid samples. Additionally, we observed a more homogeneous interaction network in the Php group, which is consistent with the findings of most studies on *H. pylori* infections.

Finally, we predicted the functional metabolism of the gastric microbiota in the three groups using microbial abundance and the KEGG database. The results showed that, compared to the Nhp group, the Php group was significantly more enriched in pathways associated with bacterial motility proteins, flagellar assembly, bacterial chemotaxis, secretion systems, oxidative phosphorylation, and epithelial cell signaling during *H. pylori* infection. In addition to sharing the same four enriched pathways as the Php group, the Rhp group exhibited upregulation of unique pathways, including propanoate metabolism, leucine and isoleucine degradation, and protein kinase signaling.

Propanoate, a short-chain fatty acid, is known to modulate gastrointestinal hormones, delay gastric emptying, prolong contact between chyme and mucosa, disrupt the mucosa’s self-repair balance, and cause cellular damage. Additionally, studies have shown that the activation of protein kinases, including JNK and p38, in gastric epithelial cells can induce mucosal cell injury (Arab et al., 2019; Jia et al., 2007). These findings suggest that RHPI is more likely than initial *H. pylori* infection to cause mucosal damage.

Our study also identified, for the first time, a higher abundance of sulfur metabolism pathways in patients with RHPI than in those with initial *H. pylori* infection. Microbiota associated with high sulfur metabolism is recognized as a risk factor for colitis (Gobert et al., 2024). Whether the upregulation of the sulfur metabolism pathway in RHPI represents a similar risk factor for gastric inflammation requires further investigation.

Recent studies using the string test method to detect *H. pylori* infection have demonstrated the utility of obtaining free *H. pylori* from gastric fluid (Wang et al., 2024). This study innovatively used gastric fluid samples for microbiota analysis, utilizing a non-invasive sampling method that is simple to perform, poses no risk to the patient, and does not require coagulation function testing. Additionally, the gastric fluid comes into prolonged contact with all parts of the gastric mucosa, likely providing a comprehensive and representative reflection of the gastric microbiota environment. The use of gastric fluid samples overcomes the limitations of gastric mucosal biopsy, such as invasiveness, small sample size, and difficulty in repeated sampling within a short period. This method holds a considerable potential for widespread application in large-scale microbiota studies of other gastric diseases and in the monitoring of gastric microbiota. However, our study was limited by a relatively small sample size and its cross-sectional design, which precludes definitive conclusions regarding causal relationships between variables. Although the 16S rDNA sequencing and PICRUSt2 predictions in this study provide valuable insights, further validation through metagenomic analysis or targeted qPCR is needed.

In summary, this study innovatively used gastric fluid samples for microbial profiling and functional analysis, revealing distinct microbiota features in patients with RHPI. We further explored potential mechanisms underlying disease progression, thereby offering important theoretical support for the development of more effective therapeutic strategies.

## Data Availability

The original contributions presented in the study are publicly available. This data can be found here: https://www.ncbi.nlm.nih.gov, accession number PRJNA1279804 (BioProject ID: 1279804).
