# Supplementary figures and images for: Characteristics of gastric fluid microbiota in patients with refractory Helicobacter pylori infection
Source: Front Microbiol. 2025 Jul 1;16:1618803. doi: 10.3389/fmicb.2025.1618803 (PMC12261676; doi:10.3389/fmicb.2025.1618803)

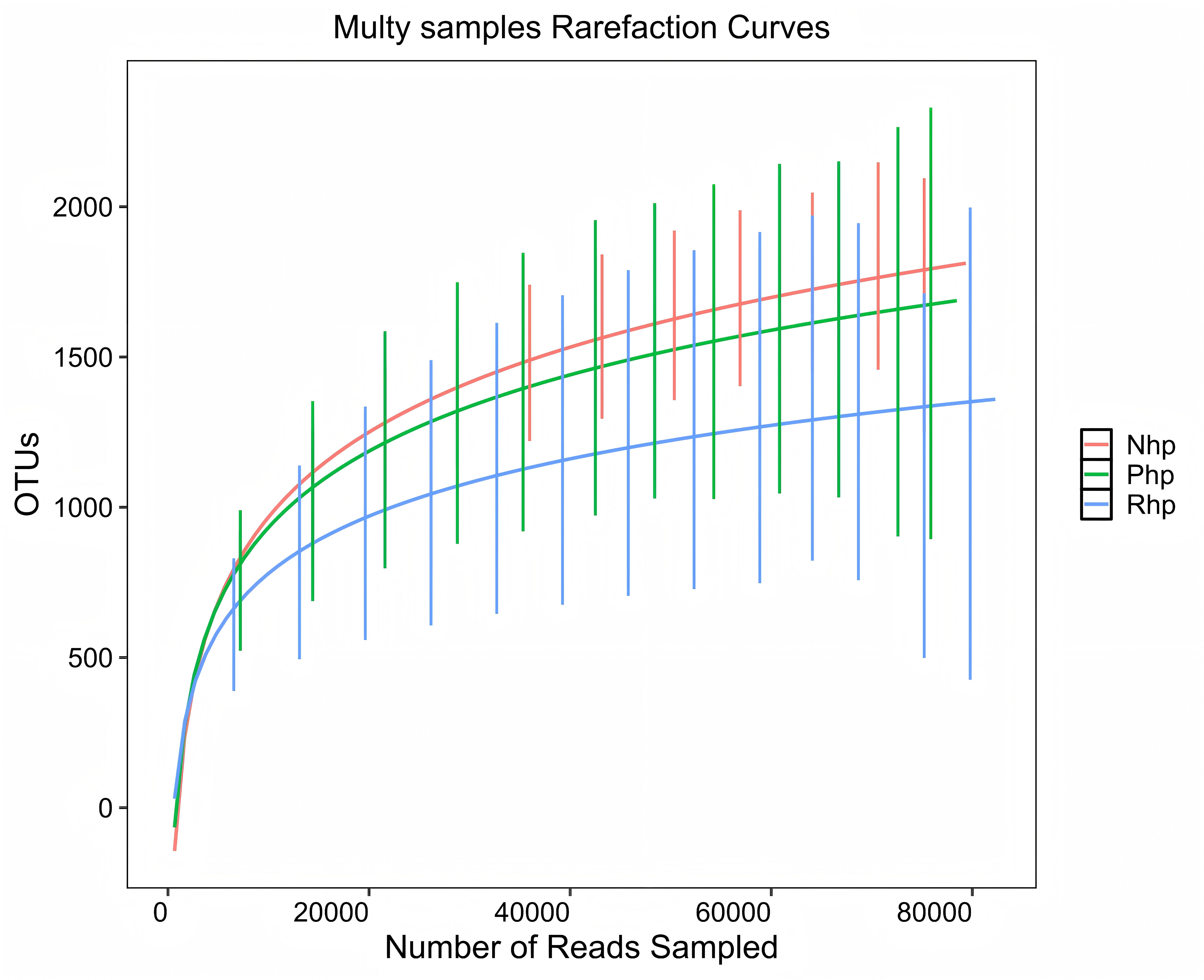

Supplement: Supplementary file 1 [file Image_1.jpeg]

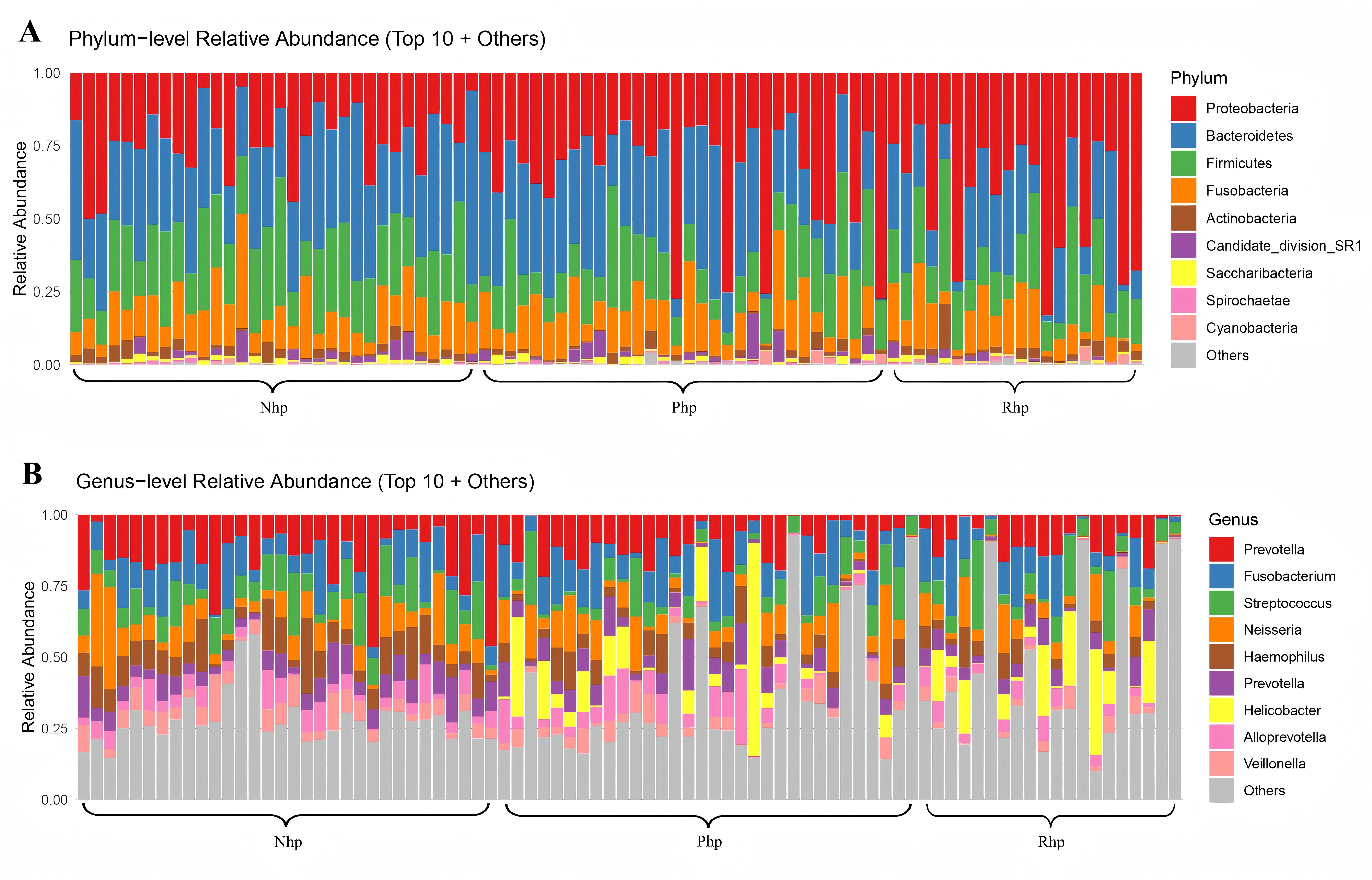

Supplement: Supplementary file 2 [file Image_2.jpeg]
